# Considerations for planning COVID-19 treatment services in humanitarian responses

**DOI:** 10.1186/s13031-020-00325-6

**Published:** 2020-11-25

**Authors:** Sylvia Garry, Nada Abdelmagid, Louisa Baxter, Natalie Roberts, Olivier le Polain de Waroux, Sharif Ismail, Ruwan Ratnayake, Caroline Favas, Elizabeth Lewis, Francesco Checchi

**Affiliations:** 1 Health in Humanitarian Crises Centre, Department of Infectious Disease Epidemiology, London School of Hygiene & Tropical Medicine, Keppel Street, London, WC1E 7HT UK; 2 Save the Children UK, 1 St John’s Ln, Farringdon, London, EC1M 4AR UK; 3 Centre de réflexion sur l’action et les savoirs humanitaires (CRASH), Fondation MSF (Médecins Sans Frontières), 13-34 avenue Jean Jaurès, 75019 Paris, France; 4 UK Public Health Rapid Support Team (UK-PHRST), London School of Hygiene & Tropical Medicine, Keppel Street, London, WC1E 7HT UK; 5 Department of Global Health and Development, Department of Infectious Disease Epidemiology, London School of Hygiene & Tropical Medicine, Keppel Street, London, WC1E 7HT UK

**Keywords:** COVID-19, SARS-CoV-2, Coronavirus, Africa, Low-income, Humanitarian, Crisis, Fragile, COVID-19 treatment service delivery, Treatment

## Abstract

The COVID-19 pandemic has the potential to cause high morbidity and mortality in crisis-affected populations. Delivering COVID-19 treatment services in crisis settings will likely entail complex trade-offs between offering services of clinical benefit and minimising risks of nosocomial infection, while allocating resources appropriately and safeguarding other essential services. This paper outlines considerations for humanitarian actors planning COVID-19 treatment services where vaccination is not yet widely available. We suggest key decision-making considerations: allocation of resources to COVID-19 treatment services and the design of clinical services should be based on community preferences, likely opportunity costs, and a clearly articulated package of care across different health system levels. Moreover, appropriate service planning requires information on the expected COVID-19 burden and the resilience of the health system. We explore COVID-19 treatment service options at the patient level (diagnosis, management, location and level of treatment) and measures to reduce nosocomial transmission (cohorting patients, protecting healthcare workers). Lastly, we propose key indicators for monitoring COVID-19 health services.

## Background

### The challenge of treating COVID-19 in humanitarian responses

COVID-19 epidemics are resulting in high excess morbidity and mortality across high-income countries. The virus is expected to cause even more pernicious effects in crisis-affected populations, defined here as forcibly displaced people within or across national borders and non-displaced persons affected by armed conflict, exceptional food insecurity and/or natural disasters, and in need of humanitarian assistance. These populations may face higher COVID-19 attack rates due to large household sizes, inadequate hygiene and access to safe water and sanitation, and camp or urban overcrowding; they may also experience higher disease severity and poorer outcomes due to untreated co-morbidities and limited access to health services [1].

COVID-19 treatment services may prove particularly challenging in settings with low baseline healthcare capacity, fragile supply chains and limited access to testing. Aside from the complexities of adapting clinical protocols to these conditions, humanitarian actors are likely to face complex trade-offs when deciding whether and which COVID-19 health services can be offered in a given setting. In this paper, we outline considerations and decision-making criteria for humanitarian and government actors to adapt routine health services and design COVID-19 health services. While the World Health Organization (WHO) has published clinical management guidelines [2, 3] and ethical guidance for supporting decision-making in outbreaks [4], we focus here on supporting decision-makers in balancing resource allocation across the spectrum of population healthcare needs until vaccination is widely locally available.

### Principles and objectives of COVID-19 treatment services

We suggest that the following principles should underpin decision-making and planning for provision of COVID-19 health services:

- ▪ Beneficence: Care offered, particularly outside the home and in settings where patients are separated from their families, should offer an **evidence-based clinical benefit** where available (including documented, publicly-available clinical experience) to the type of patient for whom it is intended – for example, critical cases, severe but non-critical cases, or non-severe with known risk factors [3]. Accordingly, COVID-19 inpatient facilities should admit patients whose severity profile they are equipped to mitigate, which will depend on resource availability and expertise / experience (see Table 1). The most experienced clinicians available should make this assessment at triage;
- ▪ Non-maleficence: COVID-19 health services must keep to a minimum the risk of **nosocomial SARS-CoV-2 infection** for clinical and support staff. Proposed COVID-19 treatment services should not be pursued if this risk could (i) present staff with a dilemma between caring for patients and preserving their health, particularly when abstaining from care provision would result in loss of income or stigma [11]; (ii) cause unacceptable absenteeism, mortality or long-term disability among healthcare workers, particularly where such losses would leave serious, long-term gaps in non-COVID-19 health service delivery [12, 13]; and/or (iii) propagate transmission within healthcare settings (e.g. to non-COVID-19 patients) to an extent likely to negate the clinical benefits of treatment.
- ▪ Justice – efficiency: Against finite resources, COVID-19 treatment services must be carefully balanced to not excessively withdraw resources from potentially more **cost-effective interventions** [14] to mitigate both the direct effects of the epidemic (such as non-pharmaceutical prevention) and its indirect effects due to disruption of essential routine health services;
- ▪ Justice – equity of resource allocation: If COVID-19 treatment service capacity is not sufficient to meet demand, it should be offered equitably, with priority attributed to patients who would be **most likely to benefit** from treatment or palliation. Corresponding triage and admission criteria should be communicated and understandable to the community;
- ▪ Justice – equity of access: COVID-19 health services should be designed to **proactively** **address barriers to accessing care **by those most in need and should be accountable and acceptable to the catchment population, with an emphasis on dialogue and transparent communication.

**Table 1:** Options for health services for confirmed or suspect COVID-19, by level of the health system

Level of care	COVID-19 treatment services objectives	Interventions
**Home / community**	Promote safe and dignified home care Reduce intra-household, community and nosocomial transmission	• Promote home (and, if possible, self-) care of non-severe COVID-19 symptoms through supportive treatment (e.g. antipyretics) [5], adequate hydration and nutrition. This can be supported through community messaging and training of healthcare workers; • Undertake risk communication and behaviour change promotion to limit transmission within households and the wider community (through patient home isolation and household self-quarantine), and to make patients aware of when to seek higher levels of care (e.g. for worsening symptoms); • Identify people with risk factors for severe COVID-19, advising them on care-seeking and promoting earlier supportive treatment if COVID-19 symptoms occur (e.g. antipyretics) [5]; • Involve community health workers (CHWs) in COVID-19 treatment service delivery, appropriate to their current workload and skillset, as a secondary priority after COVID-19 risk communication and behaviour change promotion [6]: CHW involvement may include advice on home care, treatment seeking and self-isolation; identification, monitoring and advice to people at high-risk of severe COVID-19; support for home based palliative care [7] and delivery of drugs and supplies to reduce patients’ need to visit health facilities; • Follow up for high-risk patients discharged from inpatient care who could develop/ have developed complications (e.g. poor nutritional status, respiratory difficulties).
**Outpatient care**	Promote safe and dignified home care Identify patients in need of hospitalisation	• Encourage home care of non-severe cases, as above; • Identify suspect COVID-19 patients with signs and symptoms of severe illness and refer them onward if higher-level care is available; • Treat co-morbidities and co-infections, e.g. malaria, that may be complicating the clinical picture; • Identify people with risk factors for severe COVID-19 [3] and assess presence of complications including hypoxia or respiratory distress. Consider these patients for early hospital admission (if appropriate and where available) e.g. to facilitate monitoring and maintenance of oxygen levels. If they are well but at high risk, consider monitoring them in the community (if feasible and safe for CHWs) [6, 8]; • Follow up for high-risk patients discharged from inpatient care who could develop/ have developed complications.
**Inpatient (district hospital, in a context where no respiratory support, e.g. oxygen, is available)**	Manage some COVID-19 complications Identify patients in need of more advanced care	If oxygen is not available, the risks of inpatient care are likely to outweigh the benefits. However, worthwhile interventions may include: • Identify people with risk factors for severe COVID-19 [3] and assess presence of complications including hypoxia, respiratory distress, sepsis, dehydration, poor blood sugar control in diabetics, hypertension, and co-infections; and manage complications to the extent possible; • Consider these patients for onward referral (if appropriate and where available) to facilitate monitoring and maintenance of oxygen levels and management of other complications; • Offer palliative care if no further escalation of care is available or appropriate [7].
**Inpatient (more advanced care including non-invasive respiratory support)**	Supportive care to improve clinical outcomes	As above plus: • Offer basic respiratory support (e.g. oxygen) as per COVID-19 clinical guidance [3]; • Offer other means of non-invasive ventilation, e.g. continuous positive airway pressure [9] if its effectiveness is confirmed, and with consideration for possible associated risk of nosocomial transmission [10]; • Offer palliative care (as above) if supportive therapy is unsuccessful.
**Inpatient (advanced intensive care)**	Intensive care to improve clinical outcomes	As above plus: • Manage critical cases through supportive measures including invasive ventilation, cardiovascular support and renal supportive care.

In accordance with the above principles, we suggest that COVID-19 health services in humanitarian responses should be designed to achieve *all* of the following objectives:

1. Safeguard the delivery of essential non-COVID-19 health services;
2. Protect frontline healthcare and support workers from infection;
3. Allocate resources optimally and equitably, while minimising opportunity costs (e.g. diversion of resources from more cost-effective interventions);
4. Reduce COVID-19 case-fatality and morbidity through safe, dignified and effective COVID-19 health services including palliative care where appropriate.

## Decision-making and resource allocation

**Fig. 1.**
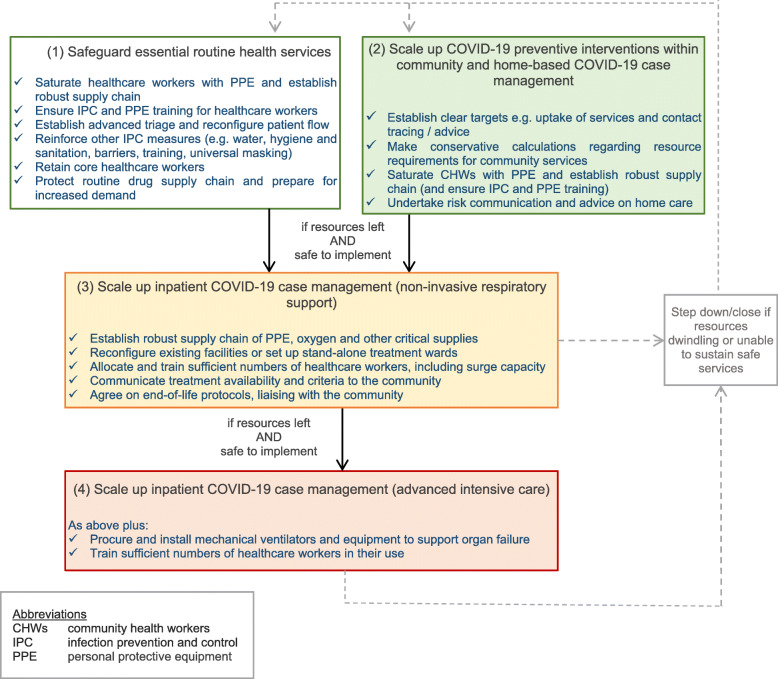
Suggested decision-making flowchart to support decision-makers responding to the COVID-19 pandemic in humanitarian health settings

We propose a structured approach, coordinated across humanitarian actors, local health authorities and communities, to take decisions on which package of COVID-19 health services is appropriate locally [15]. Factors for consideration are summarised below. Figure 1 outlines an approach for decision-making: in brief, we suggest that COVID-19 health services at facility level (3 and 4 in Fig. 1) should only be considered as a third priority, if resources are still available after securing essential health services (1 in Fig. 1) and enacting COVID-19 preventive measures within community and home-based COVID-19 case management (2 in Fig. 1).

### Community preferences

Decisions on which COVID-19 health services to offer should be accompanied by proactive, ongoing dialogue with legitimate community members representing a plurality of interests and perspectives [16, 17]. Humanitarian actors should communicate the rationale for resource allocation and changes to services; they should, however, also be prepared to adapt blueprints to the preferences of the local community (e.g. around burial, end-of-life care or home- versus facility-based care for severe cases, where oxygen is unavailable); or support appropriate community innovations (e.g. around home care). Community dialogue may also improve service design and utilisation (e.g. by identifying barriers to accessing care).

### Rational allocation of resources

#### Mitigating direct versus indirect morbidity and mortality

The economic trade-offs of mitigating the direct and indirect consequences of COVID-19 are complex and only beginning to be quantified. While it may be rational to withdraw resources from some routine health services to scale up COVID-19 health services, emerging evidence suggests that the pandemic could cause severe disruptions to disease control programmes for tuberculosis, HIV and malaria, causing indirect mortality on a scale comparable to the epidemic itself [18], as noted during the West Africa Ebola epidemic (2013–2016) [19], as well to routine vaccination services [20]. Moreover, when considering a metric of disability-adjusted life years lost, the comparison between routine health services (which disproportionately benefit younger age groups) and COVID-19 care (benefiting older age groups) is likely to favour the former even more than crude mortality.

On balance, we believe therefore that preserving routine, essential health services is a more appropriate use of limited resources than scaling up COVID-19 health services to provide more intensive inpatient hospital care (Table 1) [21]. Modelling studies suggest the benefits of maintaining routine vaccination and tuberculosis services during the pandemic far outweigh the risks of nosocomial transmission of COVID-19 among people seeking such routine services in health centres [22, 23]. Preserving the functionality and accessibility of a locally-defined package of essential health services (e.g. integrated management of childhood illness, management of acute malnutrition, vaccination, family planning, antenatal care, management of obstetric and neonatal emergencies, management of non-communicable diseases, tuberculosis and HIV treatment, trauma surgery and vector control) should thus take first priority in the public health response to the pandemic. This includes preferentially directing scarce Personal Protective Equipment (PPE) supplies to these services, and not diverting core healthcare workers to COVID-19 health services or triage, or to public health and surveillance functions that can be fulfilled by non-clinical staff.

#### Prevention versus treatment

The opportunity costs of increasing COVID-19 health services should also be considered in terms of foregoing non-pharmaceutical interventions to reduce the epidemic’s impact, to the extent that the two sets of interventions may compete for the same financial, human and material resources. Relatively cheap preventive measures such as behaviour change promotion [8], water, hygiene and sanitation improvements, use of face masks, and shielding of elderly or high-risk persons could achieve considerable reductions in COVID-19 mortality [24], and reduce pressure on health services. Conversely, insufficient prevention would likely result in a demand for hospitalisation capacity far in excess of even optimistic scale-up assumptions [25].

#### Population- and patient-level prioritisation

When demand outstrips capacity, allocation of resources must be based on equity, effectiveness and cost-effectiveness considerations. At the population level, this means geographically locating care closest to those most vulnerable, e.g. communities with the least ability to adopt COVID-19 preventive measures and/or the highest prevalence of known COVID-19 risk factors (e.g. untreated non-communicable diseases [3]). Conversely, at the patient level, whilst considering community preferences, priority should be given to patients most likely to benefit from care, as per explicit, transparent clinical decision-making criteria based on prognostic indicators and vulnerability scoring.

In extreme scenarios where capacity is insufficient even for the highest-priority patient groups, decision support frameworks similar to those used for mass casualty incidents and disaster response may need to be temporarily applied [26] and should be re-evaluated frequently. All patients should receive compassionate care including symptomatic relief. Frameworks for these decisions should be discussed with the community at the early stages of the outbreak and, ideally, with other actors such as the Ministry of Health to ensure equity and transparency.

### Risks and minimum requirements for inpatient care

In most settings, short of a prolonged lockdown, modelling projections suggest that baseline hospitalisation capacity may need to be increased by 10–1000 fold [27]. Designing COVID-19 health services accordingly requires information on:

- Reasonable projections of peak expected caseload, by severity (non-severe cases are also important for planning, as they may greatly increase demand for outpatient care). Scenarios should be conservative, with the flexibility to be adjusted according to real-time observations;
- Realistic capacity to scale up and sustain key inputs including hospital infrastructure (e.g. electricity supply, water, sanitation and hygiene), healthcare workers (reallocation and training), treatment supplies, and PPE [28]. The WHO has developed tools to support resource quantification [29]; in particular, high volumes of oxygen are difficult to ensure if only oxygen concentrators are available.
- Health service resilience, meaning its capacity to adapt to meet the healthcare needs of the population which requires dependable and adaptable resources including supply chains, reliable funding for healthcare, and reserve healthcare worker capacity (loss of healthcare workers due COVID-19 infection or fear of acquiring the infection should be expected during the epidemic) [30, 31].

Among rate-limiting factors, healthcare workers merit special considerations. Advanced care for COVID-19 requires high ratios of appropriately qualified and trained clinicians to patients (this varies by setting and patient profile e.g. 1:1 or 1:2 for nurses in UK critical care settings [32]); when these ratios are diluted, risks of low-quality care and harm to patients and healthcare workers increase. At the community, triage or outpatient level, additional capacity could be sourced by mobilising networks of allied health professionals (e.g. HIV or TB community outreach), volunteers (e.g. Red Cross / Red Crescent) and professionals from other sectors (e.g. teachers). Psychological support for staff is imperative to address trauma from capacity limitations or high case-fatality, both of which may also increase the risks of attacks on healthcare workers.

It is important to consider that the risks of providing poor-quality care could be as great as the risks of not providing care at all, either scenario leading to stigmatisation of local healthcare workers, community resistance, deteriorating relations with beneficiaries, and morbidity and mortality from COVID-19 and other preventable deaths. The potential for harm to healthcare workers due to COVID-19 should be an overriding concern.

## Planning COVID-19 treatment services

Table 1 summarises COVID-19 treatment service options that, given current evidence, are likely to provide some clinical benefit and/or avert harm to patients, caregivers and healthcare workers at each level of the health system. Further details on diagnosis, assessing severity, and management can be found in WHO guidance [3].

### Considerations for service delivery

#### Location and level of care

Patients with mild COVID-19 symptoms and no risk factors may experience equivalent and clinical benefits from appropriate home care by family members. On balance, these benefits may outweigh risks posed by treatment outside the home; for example, the risk of nosocomial transmission, spending long periods away from the family, and diversion of resources away from other cost-effective interventions [5].

Experience from middle and high-income settings suggests many severe cases require respiratory support [33], while ventilated critical cases experience case-fatality ratios > 50% [34, 35], against substantial care costs. Outpatient and inpatient care in contexts that are unable to offer even oxygen support are unlikely to offer an appreciable clinical benefit for severe cases [36]: however, since it is inevitable that patients will present, it is essential to plan care pathways even in these contexts.

On balance, therefore, we suggest that high coverage of home care for COVID-19 should be prioritised first, before allocating resources to supportive respiratory care, expanding to intensive COVID-19 care for critical cases only if lower care levels have been saturated in terms of coverage and quality. Home care, however, should be accompanied by careful messaging for non-COVID-19 illness, so that patients, in particular children and pregnant women, still seek prompt care: as discussed above, establishing triage and safe COVID-19 health services in routine health facilities would preserve patient and provider confidence and is thus part and parcel of a COVID-19 home care approach.

#### Test-confirmed versus syndromic diagnosis

Testing for SARS-CoV-2 infection is generally preferable to syndromic management, if available. However, patients should receive timely and appropriate clinical care while awaiting test results (as available, see Table 1). If in short supply, tests should be preferentially reserved for healthcare workers [37] and patients in whom a SARS-CoV-2 test result would influence clinical management (i.e. by narrowing the differential diagnosis).

Where testing is not widely available (at the time of writing, we believe this applies to most humanitarian responses), syndromic management may be inevitable [38]. This approach will however be complicated by the overlap of signs and symptoms between COVID-19 and other common diseases, including acute respiratory infections (ARI) and malaria. The frequency of presenting syndromes that fit both COVID-19 and other illnesses as possible diagnoses will be greatest as the COVID-19 epidemic peaks, in specific age groups (e.g. children), geographic settings, or seasons with a high background incidence of ARI and other acute illnesses. It follows that syndromic case management of suspect COVID-19 cases will need to simultaneously combine:

1. A standard case definition, based on the latest evidence available; and
2. Presumptive management of COVID-19, assuming that the patient is indeed sick with COVID-19, as per Table 1; and
3. Presumptive management of other possible diagnoses, where possible ruling out diagnoses through available diagnostics (e.g. rapid malaria tests), and offering care for other illnesses as per signs and symptoms. Syndromic case management should holistically manage patients according to their presentation, and not solely focus on COVID-19; an overly vertical approach would likely result in excess mortality due to untreated non-COVID-19 health problems.

In Table 1, concurrent management of non-COVID-19 syndromes is implied as a default option. Such case management should follow local pre-pandemic service specifications, with adaptations to reduce nosocomial transmission of SARS-CoV-2 (see below).

#### Isolation without treatment

Simple isolation of cases in hospitalisation wards or other structures, if no treatment is being offered, is likely to have only a marginal effect on reducing transmission (due to the large proportions of asymptomatic cases [39]), could rapidly exceed capacity of available structures, would divert limited resources from more effective interventions, may deter people from seeking healthcare, and could put individuals at risk in conflict settings. Self-isolation at home is likely to be more impactful and acceptable [5]; as such we do not consider isolation without treatment further in this paper as a valid intervention.

#### Experimental therapy

The use of experimental therapy should be discouraged except under clinical trial or monitored emergency use conditions, as these may be ineffective or even harmful, and can divert resources away from cost-effective interventions such as inpatient nutritional support or early rehabilitation [3].

#### Palliative care

Palliative care may be delivered in the home or hospital depending on bed capacity, cultural appropriateness, patient preference and availability of community health services. Patients must receive compassionate, dignified end-of-life care [3, 7]; high-intensity resources and equipment should be reserved for patients more likely to benefit clinically. Visitation by selected low-risk relatives could be facilitated where possible, to not only support psychosocial wellbeing but also potentially to provide care; however these must be carefully balanced against the risk of nosocomial spread and onward spread in the community.

### Preventing nosocomial transmission

**Fig. 2.**
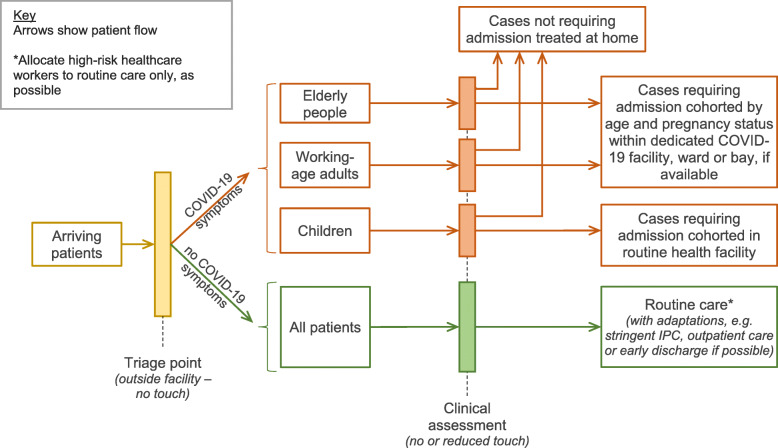
Possible configuration of patient pathways and cohorting within a routine primary- or secondary-level facility during a period of high SARS-CoV-2 transmission and in a setting with high background incidence of other diseases with similar symptoms

Table 2 lists measures to reduce nosocomial transmission of SARS-CoV-2 in healthcare settings. In addition to preventing infection, these measures are critical to preserve caregiver and patient confidence in health services. Figure 2 suggests a possible generic set-up for a routine health facility to triage, separate and manage suspected or test-confirmed COVID-19 and other patients.

**Table 2:** Measures to prevent SARS-CoV-2 nosocomial transmission within healthcare settings

**Health services**:	
• Communicate risk and treatment advice to the community, so as to promote early recognition of symptoms by patients and their caregivers, and informed decisions on whether and where to seek care;	
• Manage patients at home or at the outpatient level where possible and safe to do so (see Table 1);	
• Make every health service contact count: reinforce messaging on behaviour change and hygiene measures for patients and their caregivers;	
• Adopt at a minimum distancing between people where possible, universal usage of face coverings (especially where distancing between people is not possible), good ventilation and basic IPC measures (frequent hand hygiene and wearing of medical masks as appropriate) [40] to minimise the risk of asymptomatic or pre-symptomatic spread;	
• Triage all patients at all contact points, separating suspected COVID-19 cases from other patients [41], and adopt appropriate IPC measures [42] for any contact with suspected COVID-19 patients (see text for proposed scenarios of testing and separation). If triage and separation measures are unfeasible or overwhelmed by caseload, ensure basic IPC measures;	
• Adopt patient cohorting and separation measures to minimise mixing of COVID-19 and non COVID-19 patients (see text).	
**Healthcare workers:**	
• Ensure all healthcare workers adhere to IPC measures [42] for any contact with patients, irrespective of patients’ signs and symptoms and depending on level of exposure, and minimise physical contact (without such adaptations compromising clinical effectiveness);	
• Ensure all healthcare workers (including community health workers and non-clinical staff) monitor themselves and household contacts and immediately report COVID-19 symptoms. Staff should be supported to stay away from work while they or a member of their household is unwell. Where available and possible, they could be supported to stay elsewhere if a member of the household unwell and they are well (whilst still being required to isolate for the 14 day period);	
• Prioritise SARS-CoV-2 testing for health care workers who are have symptoms, so that they can return to work if negative (rather than self-isolate), and to identify those who need to stay away from work if positive.

#### Risk assessment of healthcare workers

In addition to training and strict infection prevention and control (IPC), morbidity and mortality among healthcare workers is likely to be reduced if staff at high risk of severe outcomes (e.g. those with co-morbidities) are preferentially allocated to routine non-COVID-19 care; those who live with high-risk family members should also either avoid direct COVID-19 care or be supported to live separately from their household.

#### Cohorting COVID-19 patients

Separating patients with COVID-19 from other patients will reduce nosocomial SARS-CoV-2 transmission, but the effectiveness of cohorting depends on diagnostic accuracy, itself a function of prevalence of true COVID-19 cases among presenting patients. Table 3 summarises qualitatively the expected positive predictive value (PPV) of COVID-19 diagnosis under alternative scenarios. If testing is available, directing COVID-19 test-confirmed patients to dedicated wards within hospitals, or to separate treatment facilities, will generally achieve clear separation from other patients, as indicated by a high positive predictive value of diagnosis.

**Table 3:** Expected variation in positive predictive value (i.e. probability that a case meeting the diagnostic criteria is truly ill with COVID-19) by COVID-19 incidence

COVID-19 incidence	Background incidence of diseases with overlapping signs and symptoms (consider the patient’s age and comorbidities, the geographic setting and season)	Modality of COVID-19 diagnosis	
		Testing	Syndromic
**High** **(around the peak)**	Low	Very high	High
	High	Very high	Low to moderate (for mild and moderate cases) Moderate to high (for severe cases)
**Medium** **(just after or just before the peak)**	Low	Very high	Moderate to high (for mild and moderate cases) High (for severe cases)
	High	High	Moderate (for mild and moderate cases) Moderate to high (for severe cases)
**Low** **(early or late in the epidemic)**	Low	High	Low to moderate (for mild and moderate cases) Moderate to high (for severe cases)
	High	Moderate to high (depends on test specificity)	Very low (for mild and moderate cases) Low (for severe cases)

By contrast, depending on the combination of COVID-19 incidence and other diseases with overlapping signs and symptoms, grouping together syndromically diagnosed COVID-19 patients is likely to expose vulnerable non-COVID-19 patients and their caregivers to nosocomial harm. To mitigate this risk, we suggest cohorting by likelihood of COVID-19 diagnosis and by risk profile [3], for example due to age or pregnancy status. Syndromically diagnosed patients should be cohorted by age (children should be cohorted separately) and pregnancy status (particularly those in the third trimester), whether in dedicated facilities or not, with restricted movement between cohorts [41]; patients known to have highly infectious co-morbidities, e.g. tuberculosis, should be treated separately.

Early triage and separation of patients with suspect COVID-19 symptoms from others remains important at both outpatient and inpatient level to reduce nosocomial transmission, even under syndromic diagnosis. However, this benefit could be negated if the COVID-19 patient pathway results in crowding and increased physical contact. In addition, syndromic diagnosis will miss asymptomatic but infectious COVID-19 patients who are attending for another reason. This highlights the importance of maintaining very stringent IPC within all healthcare settings, including for example universal usage of masks especially where it is not possible to maintain distancing between people [40].

## Service monitoring and review

Resource allocation and service design should be constantly re-evaluated in light of service utilisation and outcomes, patient and community feedback and the evolution of the epidemic itself. This requires real-time information on transmission in the community, health service performance (Table 4 suggests key indicators), and proactively elicited beneficiary feedback (e.g. through focus group discussions). Generally, patient outcome monitoring will be more difficult to interpret where testing is insufficient.

**Table 4:** Suggested key performance indicators for COVID-19 hospitalisation services. A weekly frequency of data collection and review is recommended

Indicator	Interpretation
Proportion of days with stock-out of an essential tracer medical item (e.g. oxygen, intravenous fluids, key PPE items)	Indicates robustness of supply chain and consequent quality and safety of care.
Average bed occupancy	As well as resource utilisation, < 100% occupancy during a period of known intense transmission may suggest barriers to access, including community concerns about the care being offered.
Proportion of arriving patients who met criteria for admission but were turned away or whose admission was delayed (by clinical status)	Indicates extent to which services meet demand.
Proportion of cases admitted, by age group and co-morbidity status	May indicate whether specific groups of patients (e.g. the most elderly or women) are not presenting for care: compare with what is expected based on data from the rest of the country or the region.
Proportion of critical cases among patients admitted	A high proportion of critical cases may indicate a delay in care-seeking.
Proportion of patients that become critical after admission	Indicates quality of non-invasive respiratory support and associated care. Compare with data from high-income settings.
Case-fatality ratio among non-critical patients	Indicates quality of non-invasive respiratory support and associated care. Compare with data from high-income settings.
Case-fatality ratio among critical patients	Indicates quality of invasive respiratory support and associated care. Compare with data from high-income settings. A high case-fatality ratio may also indicate the extent to which ventilation is safe and beneficial.
Proportion of healthcare workers utilising appropriate PPE, by role	Indicates availability, effectiveness of training, adherence to procedures and understanding of risk.
Proportion of healthcare workers who become ill with test-confirmed or syndromically diagnosed COVID-19	Indicates safety of care for healthcare workers. Compare with data from high-income settings. A high risk of illness or death in healthcare workers from COVID-19 could be a criterion for closing the facility.
Proportion of discharged patients who are happy with the care received	Indicates quality and humanity of care.

If the quality and safety of care are compromised, e.g. by high caseload pressure, inpatient COVID-19 services should be stepped down to ensure there are adequate resources for IPC and home / community care (see Table 1), by cutting back on intensive care first. Clinical teams should be supported to prioritise limited inpatient resources for cases with the greatest chances of recovery.

## Conclusion

Managing COVID-19 epidemics in fragile states and crisis-affected populations presents an unprecedented challenge for humanitarian actors, with huge competing population needs and limited resources. The most marginalised and vulnerable populations are likely to be the most affected. We have outlined some considerations for planning COVID-19 treatment services with the aim of holistically meeting population health priorities, supporting safe syndromic management strategies, and rationally and equitably allocating resources to prevent avoidable deaths, protect routine health services and ensure that services are appropriate and acceptable to the local population. The COVID-19 challenge is unprecedented and rapidly evolving. Resource allocation and service design must accordingly be reviewed continuously, with immediate adaptations if warranted and transparent dissemination of outcomes and experiences.

## Data Availability

Not applicable.

## Abbreviations

ARI: Acute respiratory infections
COVID-19: Coronavirus disease 2019
CHW: Community Health Worker
IPC: Infection prevention and control
PPE: Personal Protective Equipment
PPV: Positive Predictive Value
SARS-CoV-2: Severe Acute Respiratory Syndrome Coronavirus 2
WHO: World Health Organization

## References

[1] Dahab M vZK, Flasche S, Warsame A, Spiegel PB, Waldman RJ, Checchi F (2020). COVID-19 control in low-income settings and displaced populations: what can realistically be done?.

[2] World Health Organization (2020). Operational considerations for case management of COVID-19 in health facility and community.

[3] World Health Organization (2020). Clinical management of COVID-19.

[4] World Health Organization. Guidance for managing ethical issues in infectious disease outbreaks. Geneva: WHO. [Internet], Accessed 9 Sept 2020, Available from: https://apps.who.int/iris/bitstream/handle/10665/250580/9789241549837-engpdf?sequence=1&isAllowed=y.

[5] World Health Organization. Home care for patients with suspected or confirmed COVID-19 and management of their contacts, Interim guidance, 12 Aug 2020 World Health Organization, Geneva, [internet], accessed 15 Aug 2020, Available from: https://www.whoint/publications/i/item/home-care-for-patients-with-suspected-novel-coronavirus-(ncov)-infection-presenting-with-mild-symptoms-and-management-of-contacts.

[6] Ballard M, Bancroft E, Nesbit J, Johnson A, Holeman I, Foth J (2020). Prioritising the role of community health workers in the COVID-19 response. BMJ Glob Health.

[7] Pegg A, Palma M, Roberson C, Okonta C, Nkokolo Massamba/Koudika M, Roberts N. Providing end-of-life care in the emergency department: early experience from Médecins Sans Frontières during the Covid-19 pandemic. Afr J Emerg Med. 2020. 10.1016/j.afjem.2020.05.012 PMC7262500 32837875

[8] Wiah S, Subah M, Varpilah B, Waters A, Ly J, Ballard M, et al. Prevent, detect, respond: how community health workers can help in the fight against covid-19. BMJ Opnion, [internet, published online], Accessed 15 Aug 2019, Available from: https://blogs.bmj.com/bmj/2020/03/27/prevent-detect-respond-how-community-health-workers-can-help-fight-covid-19/ 2020.

[9] NHS England, NHS Improvement (2020). Guidance for the role and use of non-invasive respiratory support in adult patients with COVID- 19 (confirmed or suspected), version 3.

[10] Guan LZL, Zhang J, Peng W, Chen R. More awareness is needed for severe acute respiratory syndrome coronavirus 2019 transmission through exhaled air during non-invasive respiratory support: experience from China. Eur Respir J. 2020;55(3). 10.1183/13993003.00352-2020 PMC7098733 32198275

[11] Narasimhulu DMEV, Chazotte C, Bhatt D, Weedon J, Minkoff H (2015). Healthcare workers’ attitudes toward patients with ebola virus disease in The United States. Open Forum Infect Dis.

[12] Morley CP, Wang D, Mader EM (2017). Analysis of the association between millennium development goals 4 & 5 and the physician workforce across international economic strata. BMC Int Health Hum Rights.

[13] Evans DKGM, Popova A (2015). Health-care worker mortality and the legacy of the Ebola epidemic. Lancet Glob Health.

[14] Makhani L, Moran V, Sadique Z, Singh N, Revill P, Roberts B (2019). Examining the use of economic evaluations in health-related humanitarian programmes in low- and middle-income countries: a systematic review. Health Policy Plan.

[15] Abdelmagid N, Checchi F, Garry S, Warsame A. Defining, measuring and interpreting the appropriateness of humanitarian assistance. Int J Humanitarian Action. 2019. 10.1186/s41018-019-0062-y;14.

[16] Lough O, Holloway K. Covid-19: a watershed moment for collective approaches to community engagement? London: Humanitarian Policy Group, Overseas Development Institute. [Internet] Accessed 15 Aug 2020, Available from: https://www.odi.org/sites/odiorguk/files/resource-documents/covid-19_cce_briefing_note_web.pdf.

[17] World Health Organization (WHO), International Federation of Red Cross and Red Crescent Societies (IFRC), Humanitarian UNOftCo, Affairs (OCHA) (2020). COVID-19: How to include marginalized and vulnerable people in risk communication and community engagement Update #1 ( 20/04/2020).

[18] Hogan AB, Jewell B, Sherrard-Smith E, Vesga J, Watson OJ et al. Report 19 - the potential impact of the COVID-19 epidemic on HIV, TB and malaria in low- and middle-income countries. WHO Collaborating Centre for Infectious Disease Modelling, MRC Centre for Global Infectious Disease Analysis, Abdul Latif Jameel Institute for Disease and Emergency Analytics, Imperial College London, [internet], Accessed 23 May 2020, Available from: https://www.imperial.ac.uk/mrc-global-infectious-disease-analysis/covid-19/report-19-hiv-tb-malaria/. 2020.

[19] Parpia AS, Ndeffo-Mbah ML, Wenzel NS, Galvani AP (2016). Effects of response to 2014-2015 Ebola outbreak on deaths from Malaria, HIV/AIDS, and Tuberculosis, West Africa. Emerg Infect Dis.

[20] World Health Organization (WHO), Global Alliance for Vaccines and Immunizations (GAVI), United Nations International Children’s Fund. GAVI - 80 Million children at risk of disease as COVID idisrupts vaccination efforts, warn Gavi, WHO and UNICEF, 22 May 2020. [internet], Accessed 15 Aug 2020, Available from: https://www.gavi.org/news/mediaroom/least-80-million-children-risk-disease-covid-19-disrupts-vaccination-efforts.

[21] World Health Organization (2020). COVID-19: operational guidance for maintaining essential health services during an outbreak, interim guidance, 25 March 2020.

[22] Abbas K, Procter SR, van Zandvoort K, Clark A, Funk S, Mengistu T, et al. Routine childhood immunisation during the COVID-19 pandemic in Africa: a benefit–risk analysis of health benefits versus excess risk of SARS-CoV-2 infection. Lancet Glob Health. 2020. 10.1016/S2214-109X(20)30308-9, https://www.thelancet.com/journals/langlo/article/PIIS2214-109X(20)30308-9/fulltext. 10.1016/S2214-109X(20)30308-9 PMC7367673 32687792

[23] McQuaid CF, McCreesh N, Read JM, Sumner T, Houben RMGJ, White RG, Harris RC. CMMID COVID-19 Working Group. Eur Respir J. 2020. 10.1183/13993003.01718-2020, https://erj.ersjournals.com/content/early/2020/06/04/13993003.01718-2020. 10.1183/13993003.01718-2020 PMC7278504 32513784

[24] van Zandvoort K, Jarvis CI, Pearson CAB (2020). Response strategies for COVID-19 epidemics in African settings: a mathematical modelling study. BMC Med..

[25] Walker PGT, Whittaker C, Watson O, Baguelin M, Ainslie KEC et al. Report 12: The Global Impact of COVID-19 and Strategies for Mitigation and Suppression. WHO Collaborating Centre for Infectious Disease Modelling MRC Centre for Global Infectious Disease Analysis, Abdul Latif Jameel Institute for Disease and Emergency Analytics Imperial College London, [internet], accessed 23 May 2020, Available from: https://www.imperial.ac.uk/media/imperial-college/medicine/mrc-gida/2020-03-26-COVID19-Report-12.pdf. 2020.

[26] Coccolini F, Sartelli M, Kluger Y (2020). COVID-19 the showdown for mass casualty preparedness and management: the Cassandra Syndrome. World J Emerg Surg.

[27] Truelove S, Abrahim O, Altare C, Azman A, Spiegel P. COVID-19: Projecting the Impact in Rohingya Refugee Camps and Beyond. https://papers.ssrn.com/sol3/papers.cfm?abstract_id=3561565.

[28] IASC, IFRC, IOM, UNHCR, WHO. Scaling up COVID-19 outbreak readiness and response in camps and camp based settings. Geneva: WHO [Internet], Accessed 15 Aug 2020, Available from: https://www.who.int/publications/m/item/scaling-up-covid-19-outbreak-readiness-and-response-in-camps-and-camp-based-settings-(jointly-developed-by-iasc-ifrc-iom-unhcr-who).

[29] World Health Organization. Coronavirus disease (COVID-19) technical guidance: essential resource planning. Geneva : World Health Organization [Internet], Accessed on 2 May 2020, Available from: https://www.who.int/emergencies/diseases/novel-coronavirus-2019/technical-guidance/covid-19-critical-items. 2020.

[30] Sim MR (2020). The COVID-19 pandemic: major risks to healthcare and other workers on the front line. Occup Environ Med.

[31] Sokol DK (2006). Virulent epidemics and scope of healthcare workers’ duty of care. Emerg Infect Dis.

[32] The Faculty of Intensive Care Medicine and Intensive Care Society. Guidelines for the Provision of Intensive Care Services (GPICS) 2nd edition. UK guidance, Available online, Accessed 29 May 2020: https://www.ficm.ac.uk/sites/default/files/gpics-v2.pdf. 2019.

[33] Phua JWL, Ling L, Egi M, Lim C, Vasishtha Divatia J, Shrestha BR, Arabi YM, Ng J, Gomersall CD, Nishimura M, Koh Y, Du B (2020). Intensive care management of coronavirus disease 2019 (COVID-19): challenges and recommendations. Lancet Respir Med.

[34] Arentz MYE, Klaff L (2020). Characteristics and outcomes of 21 critically Ill patients with COVID-19 in Washington State. JAMA.

[35] Bhatraju PK, Ghassemieh BJ, Nichols M, Kim R, Keith R, Jerome KR (2020). Covid-19 in critically ill patients in the Seattle region — case series. N Engl J Med.

[36] Dondorp AM HM, Aryal D, Beane A, Schultz MJ,. Respiratory support in novel coronavirus disease (COVID-19) patients, with a focus on resource-limited settings. Am J Trop Med Hyg 2020;104269/ajtmh20-0283 [published online ahead of print, 2020 Apr 21], Accessed 23 May 2020 doi:10.4269/ajtmh20-0283.

[37] Grassly NC, Pons-Salort M, EPK P, White PJ, Ainslie K, Baguelin M (2020). Report 16 - role of testing in COVID-19 control.

[38] Howitt R, de Jesus GA, Araujo F, Francis J, Marr I, McVean M, MacMorran E, Rollinson V, Chung A, Wing Yip T. Screening and triage at health-care facilities in Timor-Leste during the COVID-19 pandemic. Lancet Respir Med. 2020. 10.1016/S2213-2600(20)30183-1. 10.1016/S2213-2600(20)30183-1 PMC7176383 32333858

[39] Oran DP, Topol EJ (2020). Prevalence of asymptomatic SARS-CoV-2 infection. Ann Intern Med.

[40] World Health Organization (2020). Transmission of SARS-CoV-2: implications for infection prevention precautions, Scientific brief 9 July 2020.

[41] World Health Organization (2020). Severe acute respiratory infections treatment centre: practical manual to set up and manage a SARI treatment centre and a SARI screening facility in health care facilities.

[42] World Health Organization (2020). Rational use of personal protective equipment for coronavirus disease (COVID-19) and considerations during severe shortages, interim guidance, 6 April 2020.

